# POST1/C12ORF49 regulates the SREBP pathway by promoting site-1 protease maturation

**DOI:** 10.1007/s13238-020-00753-3

**Published:** 2020-07-14

**Authors:** Jian Xiao, Yanni Xiong, Liu-Ting Yang, Ju-Qiong Wang, Zi-Mu Zhou, Le-Wei Dong, Xiong-Jie Shi, Xiaolu Zhao, Jie Luo, Bao-Liang Song

**Affiliations:** Hubei Key Laboratory of Cell Homeostasis, College of Life Sciences, Frontier Science Center for Immunology and Metabolism, Wuhan University, Wuhan, 430072 China

**Keywords:** SREBP, site-1 protease, proteolytic activation, unfolded protein response, activating transcription factor 6, mannose-6-phosphate

## Abstract

Sterol-regulatory element binding proteins (SREBPs) are the key transcriptional regulators of lipid metabolism. The activation of SREBP requires translocation of the SREBP precursor from the endoplasmic reticulum to the Golgi, where it is sequentially cleaved by site-1 protease (S1P) and site-2 protease and releases a nuclear form to modulate gene expression. To search for new genes regulating cholesterol metabolism, we perform a genome-wide CRISPR/Cas9 knockout screen and find that partner of site-1 protease (POST1), encoded by *C12ORF49*, is critically involved in the SREBP signaling. Ablation of POST1 decreases the generation of nuclear SREBP and reduces the expression of SREBP target genes. POST1 binds S1P, which is synthesized as an inactive protease (form A) and becomes fully mature via a two-step autocatalytic process involving forms B’/B and C’/C. POST1 promotes the generation of the functional S1P-C’/C from S1P-B’/B (canonical cleavage) and, notably, from S1P-A directly (non-canonical cleavage) as well. This POST1-mediated S1P activation is also essential for the cleavages of other S1P substrates including ATF6, CREB3 family members and the α/β-subunit precursor of N-acetylglucosamine-1-phosphotransferase. Together, we demonstrate that POST1 is a cofactor controlling S1P maturation and plays important roles in lipid homeostasis, unfolded protein response, lipoprotein metabolism and lysosome biogenesis.

**Electronic supplementary material:**

The online version of this article (10.1007/s13238-020-00753-3) contains supplementary material, which is available to authorized users.

## INTRODUCTION

Cholesterol metabolism is a complicated yet highly regulated process composed of biosynthesis, uptake, transport, utilization, export and esterification (Luo et al., 2020). Cholesterol biosynthesis and uptake represent the inputs and are switched on when cellular needs are unmet, whilst they are shut down when cellular needs are surpassed. Cholesterol within the cell is dynamically transported across the plasma membrane (PM) and various organelle membranes for serving as the membrane constituent, a signaling molecule and a precursor to other biologically active molecules (Luo et al., 2019). A balanced interplay of these pathways is essential for normal cellular functions and human health. Deregulation of cholesterol metabolism can lead to many disorders including cardiovascular disease, neurodegenerative disease and cancers (Ikonen, 2006; Kuzu et al., 2016; Chen et al., 2019).

One of the master regulators of cholesterol metabolism is sterol regulatory element-binding protein (SREBP) 2, which, through modulating expression of cholesterogenic enzymes and low-density lipoprotein (LDL) receptor (LDLR), governs cholesterol biosynthesis from acetyl-CoA and uptake from extracellular LDL particles. SREBP2 and the other two isoforms, SREBP1a and SREBP1c, are members of the SREBP family belonging to basic helix-loop-helix-leucine zipper transcription factors (Horton et al., 2002). SREBP is initially synthesized as a precursor protein with the N- and C-terminal ends facing the cytosol and two transmembrane segments spanning the endoplasmic reticulum (ER). Upon cholesterol depletion, SREBP and the associated SREBP-cleavage activating protein (SCAP), with the help of Cideb, rapidly translocate from the ER to the Golgi apparatus (Su et al., 2019). At the Golgi, SREBP is cleaved by site-1 protease (S1P) in the lumenal loop followed by a second cleavage by site-2 protease (S2P) within the membrane-spanning domain (Brown and Goldstein, 1999). This liberates the N-terminal fragment that enters the nucleus and activates the transcription of genes controlling cholesterol biosynthesis and uptake, thereby restoring cellular cholesterol levels.

A corollary of the SREBP activation model is that factors involved in ER exit, ER-to-Golgi transport and Golgi tethering of the SREBP precursor, as well as those in generation of the nuclear form of SREBP (n-SREBP) can critically regulate SREBP signaling. For examples, under cholesterol repletion conditions, INSIGs, ERLINs and TRC8 are induced to bind and retain the SCAP/SREBP complex in the ER, thereby inactivating the SREBP pathway (Irisawa et al., 2009; Huber et al., 2013; Brown et al., 2018). By contrast, AKT and PAQR3 positively regulate the SREBP pathway by promoting anterograde trafficking and Golgi anchoring of the SCAP/SREBP complex, respectively (Du et al., 2006; Xu et al., 2015). Compared with the above mechanisms controlling localization of the SREBP precursor, how its cleavage at the Golgi is regulated is less clear.

S1P (also known as subtilisin kexin isozyme-1) is a serine protease of the subtilisin/kexin proprotein convertase family (Seidah and Prat, 2012). The newly synthesized S1P is an inactive type I transmembrane precursor protein (pro-S1P) and requires multiple proteolytic events to become fully mature. Pro-S1P is first cleaved by a signal peptidase as it translocates into the ER lumen. This exposes the N-terminal prodomain that undergoes autocatalytic processing at RKVF^133^↓RSLK^137^↓, RRAS^166^↓ and RRLL^186^↓ sequentially (Cheng et al., 1999; Espenshade et al., 1999; Elagoz et al., 2002; da Palma et al., 2014). The cleaved prodomain fragments remain associated with the rest of protein to assist correct folding while retaining enzymatic activity (da Palma et al., 2014; da Palma et al., 2016). Of all three S1P forms generated, only the completely processed protein can reach the Golgi where it acts on the SREBP precursor (Sakai et al., 1998; Espenshade et al., 1999). Despite the understanding of S1P autoprocessing, little is known whether and how, if any, this process is regulated.

In the present study, we use a genome-wide CRISPR/Cas9 knockout (KO) screen to search for new regulators involved in cholesterol homeostasis. An uncharacterized gene *C12ORF49* is found to be tightly correlated with the lower PM cholesterol level in our screen. We further demonstrate that C12ORF49 interacts with S1P and affects cholesterol metabolism by promoting S1P maturation. Hence, we rename C12ORF49 the partner of site-1 protease (POST1) to reflect its biological function. Depletion of POST1 reduces S1P-mediated proteolytic cleavage of SREBP2 and other S1P substrates. These results reveal POST1 as a newly identified factor of the SREBP pathway and S1P maturation.

## RESULTS

### Genome-wide screen identifies that POST1 regulates cholesterol homeostasis

**Figure 1:**
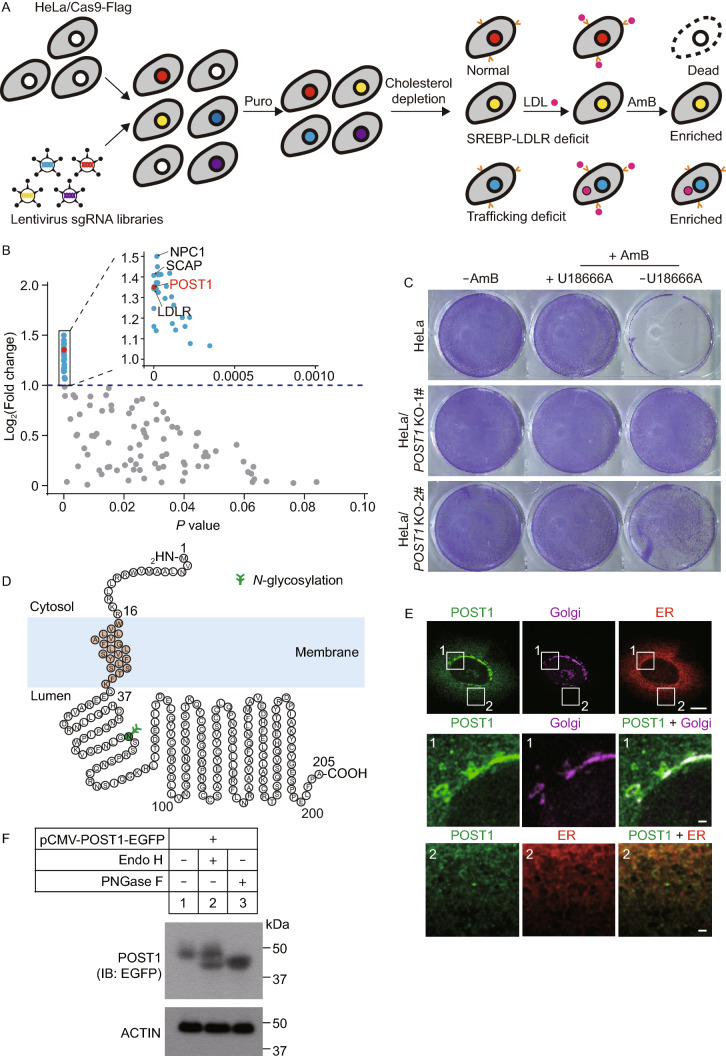
**Genome-wide screen identifies that POST1 is involved in cholesterol metabolism.** (A) Schematic representation of the screening strategy. HeLa cells stably expressing Cas9-Flag were transduced with lentivirus expressing a genome-wide sgRNA library and then treated with puromycin (Puro) for 4 days. Surviving cells were depleted of cholesterol by incubating in the medium containing 5% lipoprotein-deficient serum (LPDS) plus 10 µmol/L mevalonate and 1 µmol/L lovastatin for 16 h. Cells were then incubated with 50 µg/mL LDL for 4 h followed by 300 µg/mL amphotericin B (AmB) for 1 h. AmB could bind PM cholesterol, form pores and kill normal cells. The mutant cells defective in the SREBP-LDLR axis or cholesterol trafficking were resistant to AmB because of less PM cholesterol. After five rounds of challenges, the sgRNA inserts from surviving cells and those from transduced cells prior to the first challenge were amplified and subjected to deep sequencing. (B) Scatter plot showing 115 highly enriched genes (Supplementary Material, Table S1) in (A). Genes with a phenotype value (fold change [log_2_]) >1 and *P*-value < 0.001 are in blue (except for *POST1* in red) and are shown in smaller scales of x- and y-axes (inset). Those with a phenotype value <1 are in gray. (C) HeLa cells and two lines of *POST1* KO cells generated by the CRISPR/Cas9 technique (*POST1* KO-1# and *POST1* KO-2#) were depleted of cholesterol for 16 h. Cells were then incubated in the medium containing 5% LPDS, 50 µg/mL LDL and 1 µmol/L lovastatin in the absence or presence of 2 µg/mL U18666A for 4 h, and then in 300 µg/mL AmB for 1 h. (D) The predicted topology of human POST1 protein. (E) HeLa cells were transfected with pCMV-POST1-EGFP (green) and pCMV-DsRed2-KDEL (red) for 48 h, and immunostained with the antibody against GM130 (magenta). Boxed areas are shown at a higher magnification as numbered below. Scale bar, 10 µm (main), 1 µm (inset). (F) HeLa cells were transfected with pCMV-POST1-EGFP for 48 h and harvested. Lysates were treated with 10 units/μL Endo H or 5 units/μL PNGase F as indicated prior to immunoblotting

We first set out to identify new regulators of cellular cholesterol homeostasis using a genome-scale CRISPR/Cas9 knockout (GeCKO) screening strategy as depicted in Fig. 1A. Briefly, HeLa cells were transduced with lentivirus expressing the Streptococcus pyogenes *Cas9* gene fused to a Flag tag to generate HeLa/Cas9-Flag stable cell line. The stable cells were then transduced with lentivirus expressing a pooled GeCKO v2 library containing 65,383 sgRNAs targeting 19,050 human genes at 0.3 multiplicity of infection (Sanjana et al., 2014). A four-day puromycin selection followed to allow the untransduced cells to be all killed. Surviving cells were deprived of cholesterol by incubating in the cholesterol-depletion medium containing lipoprotein-deficient serum plus lovastatin for 16 h. This condition activates the SREBP pathway so that LDLR expression is highly induced (Goldstein and Brown, 2009). Cells were then exposed to LDL and treated with amphotericin B (AmB), an antibiotic that binds cholesterol in the PM, forms pores and causes cell death (Wei et al., 2017).

We reasoned that the cells with normal SREBP activation and cholesterol trafficking machineries could upregulate LDLR expression, take up exogenous LDL and rapidly redistribute cholesterol towards the PM by several mechanisms (Chu et al., 2015; Infante and Radhakrishnan, 2017; Luo et al., 2017, 2019; Xiao et al., 2019), thereby failing the subsequent AmB selection due to PM leakage induced by AmB. By contrast, cells defective in either SREBP–LDLR axis or cholesterol trafficking had less PM cholesterol and were resistant to AmB treatment.

To ascertain that AmB selection was stringent enough to separate defective cells from the normal ones without inducing general cytotoxicity, we subjected untransduced HeLa/Cas9-Flag cells to a parallel “cholesterol depletion-repletion-AmB selection” challenge except that U18666A, which binds NPC1 and blocks lysosomal cholesterol export (Lu et al., 2015), was added together with LDL. Cells without AmB exposure and those treated with AmB alone were used as controls. Indeed, AmB-induced cell death was effectively rescued by U18666A (Fig. S1A).

After 5 rounds of challenges, the sgRNA inserts from surviving cells and those from transduced cells prior to the first round of challenge were amplified and subjected to deep sequencing. Candidate genes were identified using MAGeCK (Li et al., 2014). Those with at least 2 gRNA hits were selected and ranked by LFC (log_2_ fold change). The LFC cutoff value was set to >0.

A total of 115 genes were found highly enriched in the cells survived 5 rounds of challenge (Fig. 1B; Table S1), among which included *NPC1*, *SCAP* and *LDLR*, the well-established regulators of cholesterol trafficking and metabolism. Specifically, loss of *NPC1* causes cholesterol accumulation in lysosomes whilst loss of *SCAP* impairs activation of the SREBP pathway. LDLR is a gatekeeper of LDL uptake as well as a target of SREBP2. Intriguingly, we also detected robust enrichment of an uncharacterized gene *C12ORF49*, to which we referred as partner of site-1 protease (*POST1*) owing to its functional association with S1P (See below).

To confirm that POST1 is indeed a critical factor for cholesterol homeostasis, we generated two independent *POST1* KO cell lines using the CRISPR/Cas9 technique followed by the cholesterol depletion-repletion-AmB selection challenge in the presence or absence of U18666A. Compared with wild-type (WT) cells that survived only when U18666A was added to AmB, *POST1* KO cells showed markedly improved resistance to AmB even in the absence of U18666A (Fig. 1C). Overall, these results support a positive role of POST1 in regulating PM cholesterol level.

Human POST1 is a small protein of 205 amino acids. It is predicted to contain a cytosolic segment (1–16 aa), a transmembrane segment (17–36 aa), and a stretch of 169 amino acids extending into the lumen (Fig. 1D). To determine the subcellular location of POST1, HeLa cells were transfected with the plasmids encoding enhanced green fluorescent protein (EGFP)-tagged POST1 and DsRed-tagged KDEL (Lys-Asp-Glu-Leu), an ER retention motif, and then immunostained with the Golgi marker GM130. We detected robust POST1 staining colocalized with GM130 and modest signal colocalized with DsRed-KDEL (Fig. 1E). POST1 contains a potential N-linked glycosylation site (Fig. 1D). The transfected POST1 protein was partially sensitive to endoglycosidase H (Endo H), but completely shifted to a lower position by peptide N-glycosidase F (PNGase F) (Fig. 1F). These results suggest that POST1 resides in both the ER and Golgi.

### POST1 is engaged in the SREBP pathway

**Figure 2:**
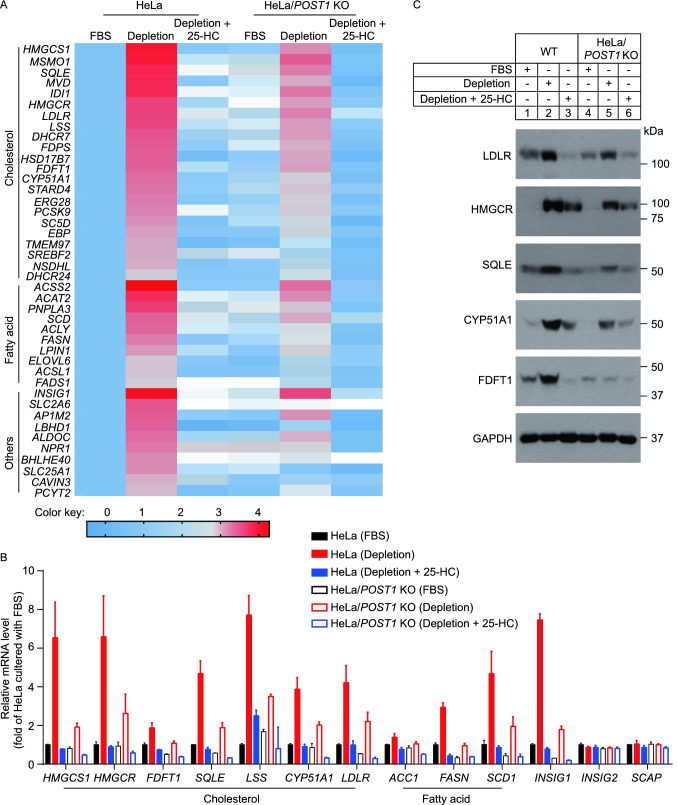
**Loss of** ***POST1*** **decreases the expression of SREBP target genes.** (A) Heat map showing gene expression profile of HeLa and HeLa/*POST1* KO cells. Cells were cultured in the medium containing 10% fetal bovine serum (FBS), or the depletion medium (5% lipoprotein-deficient serum plus 10 µmol/L mevalonate and 1 µmol/L lovastatin), or the depletion medium supplemented with 25-hydroxycholesterol (25-HC) for 16 h. The mRNA expression levels were normalized to those of HeLa cells in FBS condition. Colors indicate the gene expression range with the least expression in blue and highest expression in red. (B) Quantitative real-time PCR analysis of HeLa and HeLa/*POST1* KO cells under different culture conditions. Data were normalized to HeLa cells in FBS condition and presented as mean ± SD (*n* = 3 independent trials). (C) Immunoblot analysis of HeLa and HeLa/*POST1* KO cells under different culture conditions. ACC1, acetyl-coA carboxylase 1; CYP51A1, cytochrome P450 family 51 subfamily A member 1; FASN, fatty acid synthase; FDFT1, farnesyl-diphosphate farnasyltransferase 1; HMGCR, 3-hydroxy-3-methylglutaryl-CoA reductase; HMGCS1, 3-hydroxy-3-methylglutaryl-CoA synthase 1; INSIG, insulin-induced gene; LDLR, low-density lipoprotein receptor; LSS, lanosterol synthase; SCAP, SREBP-cleavage activating protein; SCD1, stearoyl-CoA desaturase 1; SQLE, squalene epoxidase

To gain insights into the mechanism by which POST1 governs cholesterol homeostasis, we treated WT and *POST1* KO cells with three different conditions, namely (1) normal culture medium containing fetal bovine serum (FBS), or (2) cholesterol-depletion medium, or (3) cholesterol-depletion medium plus 25-hydroxycholesterol (25-HC), and then performed whole-transcriptome-sequencing (RNA-seq). The transcriptome data of WT cells exposed to FBS was used as a reference. In both WT and *POST1* KO cells, the expression of SREBP2 target genes (cholesterol metabolism) and SREBP1c target genes (fatty acid metabolism) was markedly elevated upon cholesterol starvation (Fig. 2A; Table S2). However, these increases were much moderate when *POST1* was ablated. 25-HC as a potent inhibitor of the SREBP pathway abrogated the upregulation of lipid metabolism-related genes caused by cholesterol depletion. The expression profiles of genes involved in cholesterol and fatty acid metabolism were further verified using quantitative real-time PCR and immunoblotting (Fig. 2B and 2C). In accordance with RNA-seq results, loss of *POST1* impaired the responses of cholesterol biosynthetic genes (lanosterol synthase [*LSS*], *HMGCR*, 3-hydroxy-3-methylglutaryl-CoA synthase 1 [*HMGCS1*], squalene epoxidase [*SQLE*], cytochrome P450 family 51 subfamily A member 1 [*CYP51A1*], farnesyl-diphosphate farnasyltransferase 1 [*FDFT1*]) and fatty acid biosynthetic genes (stearoyl-CoA desaturase 1 [*SCD1*], fatty acid synthase [*FASN*], acetyl-coA carboxylase 1 [*ACC1*]), as well as those of *INSIG1* and *LDLR* to cholesterol depletion (Fig. 2B). *SCAP* and *INSIG2* are not SREBP target genes and their expression remained unaltered regardless of treatments (Fig. 2B). At the protein level, the amounts of LDLR and representative cholesterol biosynthetic enzymes were similarly increased by cholesterol depletion and decreased by 25-HC, and in a lesser extent in *POST1* KO cells (Fig. 2C).

**Figure 3:**
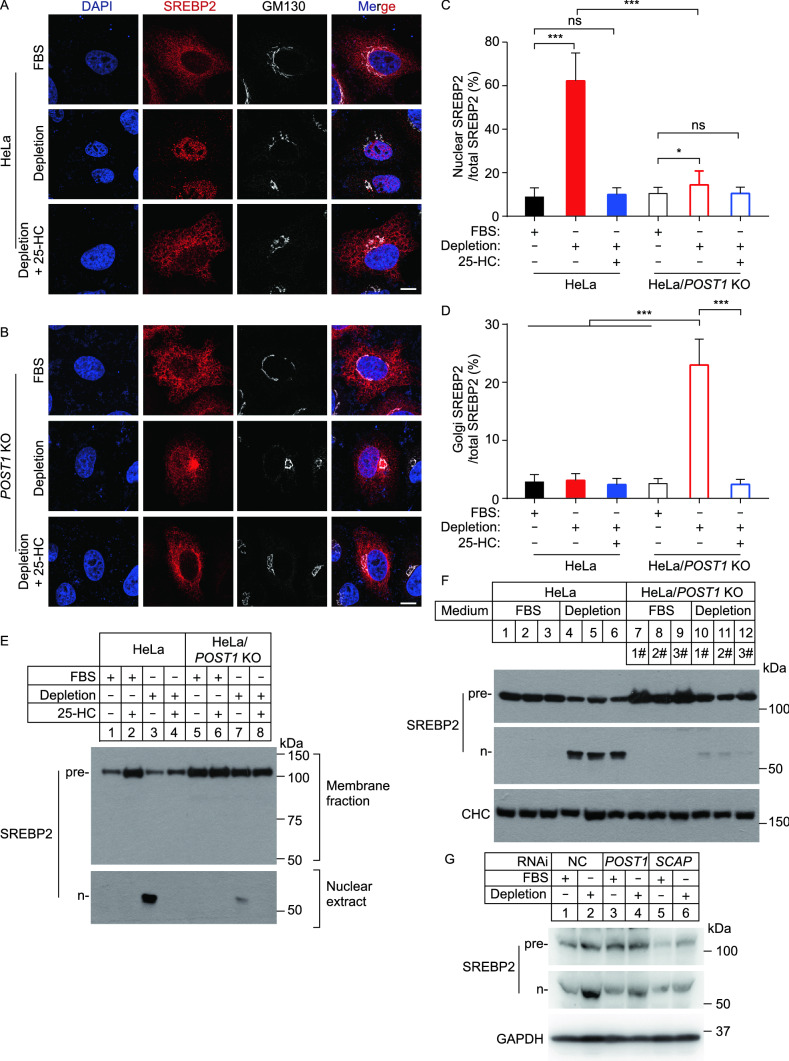
**Ablation of POST1 decreases cholesterol-depletion-induced cleavage of SREBP2.** (A and B) Confocal images showing the subcellular localization of transfected SREBP2 in HeLa (A) and HeLa/*POST1* KO (B) cells under different culture conditions. Cells were transfected with pCMV-3×Flag-SREBP2 and pCMV-SCAP for 48 h, and incubated with the indicated medium for 16 h. Cells were immunostained with the antibodies against Flag (red) and GM130 (white). Nuclei were counterstained with DAPI (blue). Scale bar, 10 μm. (C and D) Percentages of SREBP2 intensity in the nucleus (C) and Golgi (D) normalized to the total SREBP2 intensity in (A) and (B). Data are presented as mean ± SD (10 cells/trial; 3 independent trials). One-way ANOVA with Tukey HSD *post hoc* test. *P < 0.05, ***P < 0.001, ns, no significance. (E) HeLa and HeLa/*POST1* KO cells were depleted of cholesterol for 16 h and incubated with the indicated media for 16 h. Cells were treated with 25 μg/mL ALLN for 1 h prior to harvesting. Membrane fractions and nuclear extracts were prepared as described in Methods, and endogenous SREBP2 precursor was analyzed by the 1D2 antibody. Pre, precursor; n, nuclear. (F) HeLa and three different lines of HeLa/*POST1* KO cells were treated as described in (E), and whole cell lysates were subjected to immunoblotting. Pre, precursor; n, nuclear. CHC, clathrin heavy chain. (G) HeLa cells were transfected with the indicated siRNAs for 48 h and cultured in the FBS-containing or cholesterol-depletion medium for 16 h. Cells were treated with 25 μg/mL ALLN for 1 h prior to harvesting. Whole cell lysates were subjected to immunoblotting. Pre, precursor; n, nuclear

To investigate whether POST1 is directly involved in SREBP processing, we prepared a plasmid that encodes full-length SREBP2 with a Flag epitope tag at the N terminus (Flag-SREBP2). This allows tracing of SREBP2 under both cholesterol-rich and cholesterol-depleted conditions, the latter of which induces SREBP2 to liberate the N-terminal fragment (n-SREBP2) that translocates into the nucleus. WT and *POST1* KO cells were transfected with Flag-SREBP2 and challenged with varying levels of cholesterol, and the subcellular localization of SREBP2 was examined using the anti-Flag antibody. WT cells cultured in 10% FBS showed an ER distribution of SREBP2 (Fig. 3A, top row; Fig. 3C and 3D), whereas those deprived of cholesterol had robust staining in the nucleus (Fig. 3A, middle row; Fig. 3C). 25-HC blocks the ER-to-Golgi transport of SREBPs (Radhakrishnan et al., 2007), and SREBP2 was mainly found in the ER of WT cells exposed to 25-HC (Fig. 3A, bottom row). With respect to *POST1* KO cells, SREBP2 was predominately localized in the ER in the presence of ample cholesterol (Fig. 3B, top row), but redistributed to the Golgi complex with slight staining in the nucleus upon cholesterol depletion (Fig. 3B, middle row; Fig. 3C and 3D). Interestingly, 25-HC decreased both Golgi and nuclear SREBP2 staining to the levels seen in FBS-treated cells (Fig. 3B, bottom row; Fig. 3C and 3D). In line with these immunostaining results, n-SREBP2 was evident in WT cells depleted of cholesterol but barely detectible in *POST1* KO cells (Fig. 3E). We generated three independent lines of *POST1* KO cells and found markedly reduced n-SREBP2 compared with WT controls (Fig. 3F and S1B). Further, knockdown of *POST1* using small interfering RNA (siRNA) hampered cholesterol depletion-induced SREBP2 cleavage to a similar extent as knockdown of *SCAP* (Fig. 3G and S1C). Together, these results establish POST1 as a key regulator of SREBP processing.

### POST1 promotes autocatalytic cleavage of S1P

**Figure 4:**
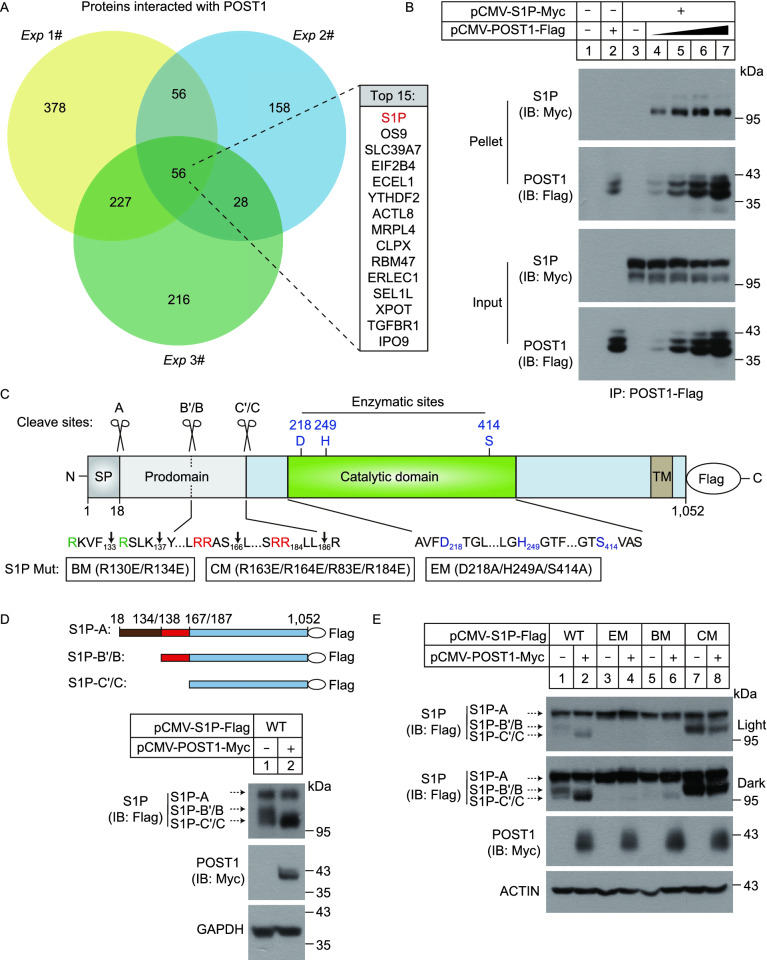

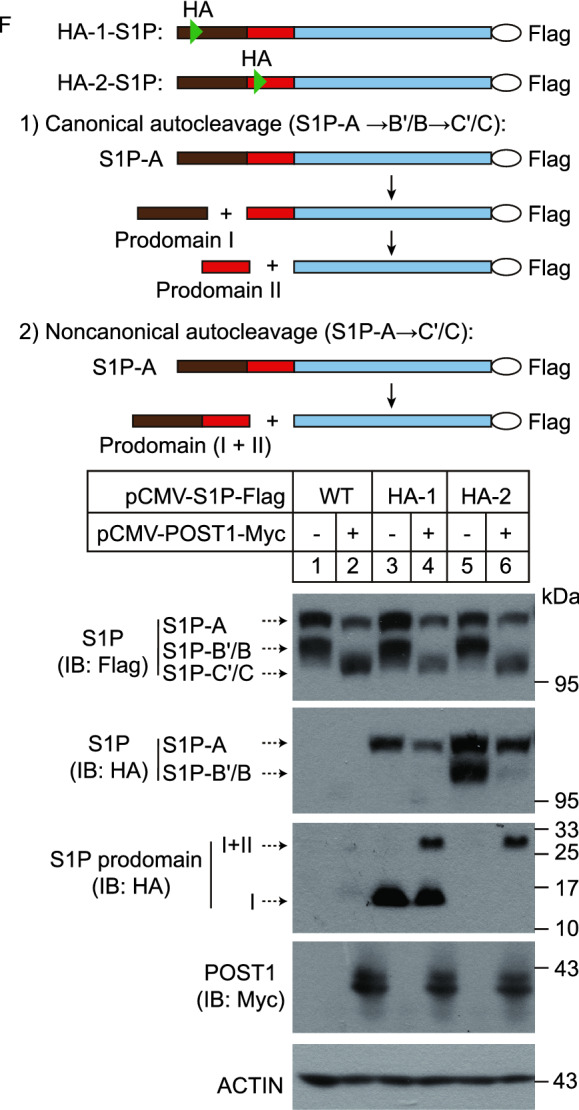
**POST1 promotes self-cleavage of S1P at the C’/C sites.** (A) Venn diagram showing POST1-interacting proteins from three independent co-immunoprecipitation experiments coupled to tandem mass spectrometry. Top 15 commonly detected protein hits were listed in details. (B) HeLa cells were transfected with pCMV-S1P-5×Myc and increasing amounts of pCMV-POST1-3×Flag as indicated for 48 h and subjected to the co-immunoprecipitation assay with the anti-Flag agarose. (C) Schematic representation of the S1P precursor with a C-terminal Flag tag. Amino acid numbers of human S1P are shown. Signal peptidase cleavage site (A), S1P autocatalytic cleavage sites (B’/B and C’/C) and enzymatic sites are indicated. SP, signal peptide; TM, transmembrane domain; BM, B’/B cleavage site mutations; CM, C’/C cleavage site mutations; EM, enzymatic site mutations. (D–F) HeLa cells were transfected with the indicated plasmids for 48 h and subjected to immunoblotting

We next sought to investigate the molecular mechanism by which POST1 regulates the SREBP pathway. HeLa cells stably expressing POST1-Flag were generated and the potential binding proteins of POST1 were identified using co-immunoprecipitation coupled to tandem mass spectrometry (MS/MS). HeLa cells stably expressing Flag alone were used as a negative control. Analysis of the MS/MS results from three independent experiments revealed a set of highly enriched proteins including S1P (encoded by *MBTPS1*) (Fig. 4A; Table S3). The POST1–S1P interaction was confirmed by the co-immunoprecipitation assay (Fig. 4B).

S1P is synthesized as an inactive precursor whose domain organization is shown in Fig. 4C. To become mature, S1P undergoes multiple processing of the N-terminal prodomain involving the catalytic triad D218/H249/S414, generating three shortened forms designated S1P-A, S1P-B’/B and S1P-C’/C (Espenshade et al., 1999; Elagoz et al., 2002; da Palma et al., 2014). Indeed, immunoblotting analysis of HeLa cells transfected with a plasmid encoding S1P fused with a C-terminal Flag tag (S1P-Flag) showed three bands corresponding to differently cleaved forms of S1P (Fig. 4D). Co-expression of POST1 increased S1P-C’/C production and eliminated S1P-B’/B (Fig. 4D). We next evaluated the impact of POST1 on processing of transfected S1P in the cells lacking the endogenous counterpart (Fig. S1D). As in the WT cells, POST1 promoted S1P-C’/C generation at the expense of S1P-B’/B as well (Fig. 4E, compare lanes 1 and 2). No S1P autoprocessing was detected when enzymatically inactive mutant (EM, D218A/H249A/S414A) was expressed alone (lane 3) or together with POST1 (lane 4), suggesting that S1P enzymatic activity is a prerequisite for its self-cleavage regardless of the presence of POST1. The B’/B cleavage site mutant (BM, R130E/R134E) of S1P failed to yield S1P-B’/B and S1P-C’/C (lane 5), whereas the C’/C mutant (CM, R163E/R164E/R183E/R184E) could generate S1P-B’/B but failed to yield S1P-C’/C (lane 7). These results are consistent with the earlier work (Espenshade et al., 1999; da Palma et al., 2014), and suggest that S1P cleavage at the B’/B sites is required for subsequent cleavage at the C’/C sites. However, we observed S1P-C’/C, albeit in small amounts, in *S1P*-KO cells co-transfected with S1P B’/B mutant and POST1 (lane 6), suggesting that POST1 may aid S1P autoprocessing bypassing the B’/B sites. By contrast, POST1 had no effect on the autoprocessing of S1P with C’/C mutations (lane 8).

It should be noted that S1P used in the above experiments had a Flag tag at the C terminus, which provided limited information on the self-cleavage steps occurring within the N-terminal prodomain. Therefore, to examine whether POST1 facilitates generation of S1P-C’/C directly from S1P-A, we prepared plasmids that encode two HA-tagged versions of S1P-Flag, designated HA-1 and HA-2. The HA epitope tag was inserted in the prodomain between A and B’/B sites (prodomain I) in HA-1, and between B’/B and C’/C sites (prodomain II) in HA-2 (Fig. 4F). S1P-Flag, HA-1 or HA-2 was transfected into HeLa cells alone or in combination with POST1, and S1P autoprocessing was analyzed by immunoblotting. The anti-Flag blot (Fig. 4F, the 1st blot) showed that POST1 increased S1P-C’/C formation when co-expressed with S1P-Flag, HA-1 or HA-2. However, in the anti-HA blot, we detected S1P-A and a 14-kDa band corresponding in size to the prodomain I in the cells transfected with the HA-1 plasmid, but A and B’/B forms in the cells transfected with the HA-2 plasmid (lanes 3 and 5 of the 2nd and 3rd blots). These results are in accordance with the canonical cleavage event in which S1P-A is converted to S1P-B’/B and then to S1P-C’/C. Theoretically, the prodomain II generated from HA-2 should also be visible, and we attribute its absence to the small protein size (~5 kDa). Upon POST1 co-expression, a band corresponding to the prodomain (I+II) appeared in the cells expressing HA-1 or HA-2 (lanes 4 and 6 of the 3rd blot). These results support the notion that POST1 promotes S1P-A cleavage at the C’/C sites. In addition, S1P-B’/B was dramatically decreased when POST1 was co-expressed (compare lanes 5 and 6 of the 2nd blot), indicating POST1 also promotes S1P-B’/B cleavage at the C’/C sites. Together, we propose that POST1 accelerates generation of mature S1P-C’/C from S1P-B’/B via a canonical cleavage as well as from S1P-A directly via a non-canonical cleavage (Fig. 6I).

**Figure 5:**
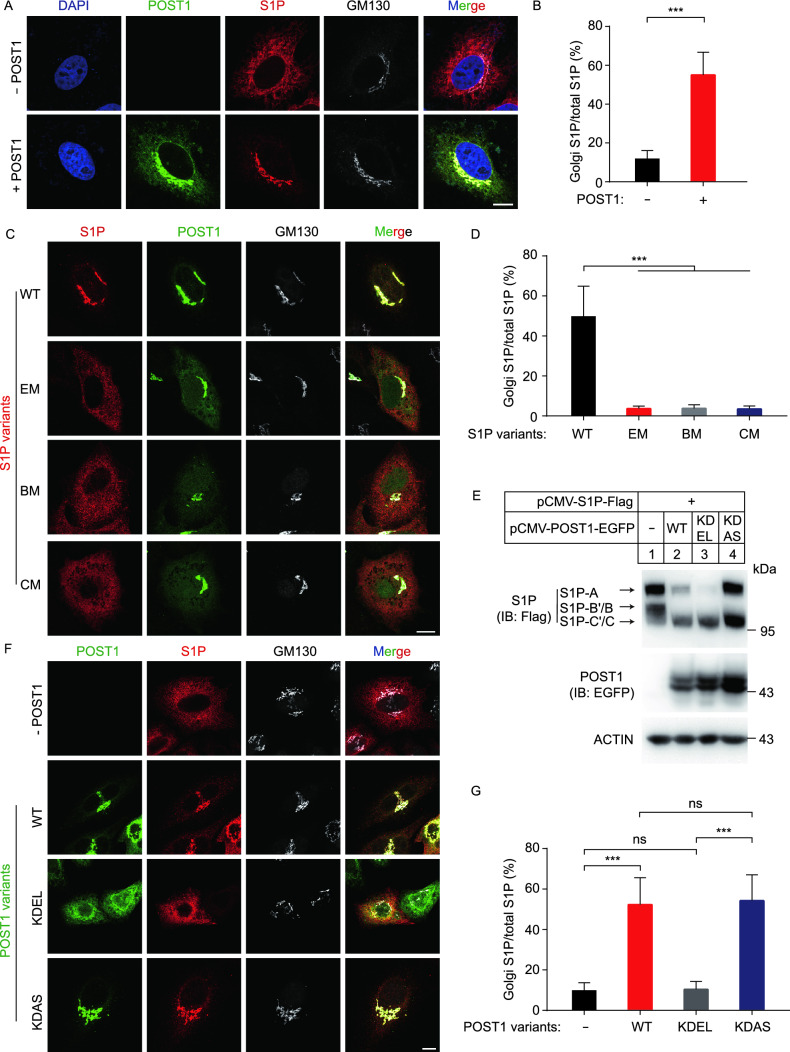
**POST1 facilitates translocation of mature S1P to the Golgi.** (A) HeLa cells were transfected with either pCMV-S1P-3×Flag (red) alone or in combination with pEGFP-N1-POST1 (green) for 48 h. Cell were fixed and immunostained with the antibodies against Flag and GM130 (white). Nuclei were counterstained with DAPI (blue). Scale bar, 10 μm. (B) Percentages of S1P intensity in the Golgi normalized to the total S1P intensity in (A). Data are presented as mean ± SD (10 cells/trial; 3 independent trials). Unpaired two-tailed Student’s *t*-test. ***P < 0.001. (C) HeLa cells were co-transfected with pCMV-POST1-EGFP (green) and pCMV-S1P-3×Flag variants (red) for 48 h. Cell were fixed and immunostained with the antibodies against Flag and GM130 (white). BM, B’/B cleavage site mutations; CM, C’/C cleavage site mutations; EM, enzymatic site mutations. The S1P variants were illustrated in Fig. 4C. Scale bar, 10 μm. (D) Percentages of S1P intensity in the Golgi normalized to the total S1P intensity in (C). Data are presented as mean ± SD (10 cells/trial; 3 independent trials). One-way ANOVA with Tukey HSD *post hoc* test. ***P < 0.001. (E) HeLa cells were transfected as indicated for 48 h and subjected to immunoblotting. (F) HeLa cells were co-transfected with pCMV-S1P-3×Flag (red) and different variants of pCMV-POST1-EGFP (green) for 48 h. Cells were fixed and immunostained with the antibodies against Flag and GM130 (white). Scale bar, 10 μm. (G) Percentages of S1P intensity in the Golgi normalized to total S1P intensity in (F). Data are presented as mean ± SD (10 cells/trial; 3 independent trials). One-way ANOVA with Tukey HSD *post hoc* test. ***P < 0.001, ns, no significance

We next investigated the effect of POST1 on S1P subcellular location using HeLa cells transfected with S1P-Flag alone or in combination with POST1-EGFP. In the absence of POST1, the majority of S1P resided in the ER and only about 12% of S1P was found colocalized with GM130 (Fig. 5A and 5B). Co-expression of POST1 caused a greater than 5-fold increase in S1P localization to the Golgi complex (Fig. 5A and 5B). Notably, mutations in enzymatic activity (EM) and B’/B or C’/C cleavage sites (BM and CM) severely impaired POST1-induced translocation of S1P from the ER to the Golgi (Fig. 5C and 5D). As these mutants (EM, BM and CM) produced little or no S1P-C’/C (Fig. 4E), it is concluded that POST1 facilitates the generation of S1P-C’/C that is subsequently transported to the Golgi. Co-expression of S1P and POST1 dramatically promoted the nuclear localization of SREBP2 (Fig. S2A; Movie S1).

To address where POST1-stimulated S1P processing occurs, we prepared plasmids encoding POST1 with a C-terminal EGFP followed by a KDEL or KDAS (Lys-Asp-Ala-Ser) tetrapeptide sequence. The KDEL tail is supposed to confer constitutive ER localization of POST1-EGFP, whereas the KDAS tail should be non-functional and serves as a negative control. To validate the localization of POST1-EGFP, POST1-EGFP-KDEL and POST1-EGFP-KDAS, lysates from cells transfected with various plasmids were treated with Endo H or PNGase F. As expected, only POST1-EGFP-KDEL was completely sensitive to Endo H, and the other two proteins were partially resistant to Endo H (Fig. S2B). These results suggest that all POST1-EGFP-KDEL proteins reside in the ER, and the other two proteins are present in both ER and Golgi. We transfected the plasmids encoding S1P-Flag and different versions of POST1-EGFP into HeLa cells, and found that both KDEL- or KDAS-tagged POST1-EGFP could facilitate S1P-C’/C production as the WT version did (Fig. 5E). Notably, unlike the Golgi localization of S1P in the cells expressing WT and KDAS-tagged POST1-EGFP, S1P was mainly retained in the ER when co-expressed with POST1-EGFP-KDEL (Fig. 5F and 5G). These results indicate that POST1 promotes self-cleavage of S1P in the ER and that the generated S1P-C’/C still binds to POST1.

### POST1 is critical for proteolytic activation of other S1P substrates

**Figure 6:**
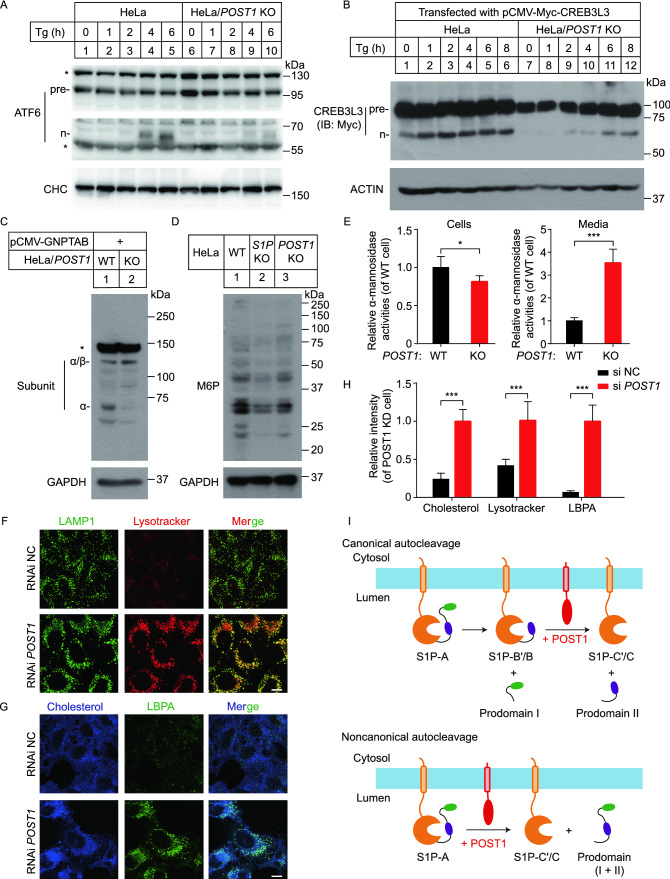
**POST1 affects proteolysis of other S1P substrates.** (A) HeLa and HeLa/*POST1* KO cells were treated with 2 μmol/L thapsigargin (Tg) for the indicated periods, and the processing of endogenous ATF6 was analyzed by immunoblotting. Pre, precursor; n, nuclear. (B) HeLa and HeLa/*POST1* KO cells were transfected with pCMV-5×Myc-CREB3L3 for 48 h and treated with 2 μmol/L thapsigargin for the indicated periods. The processing of transfected CREB3L3 was analyzed by immunoblotting. pre, precursor; n, nuclear. (C) HeLa and HeLa/*POST1* KO cells were transfected with pCMV-GNPTAB mini-construct for 48 h, and the processing of transfected α/β-subunit precursor was analyzed by immunoblotting. (D) Immunoblot analysis of HeLa, HeLa/*S1P* KO and HeLa/*POST1* KO cells using the anti-M6P antibody and GAPDH antibody. (E) Activity of α-mannosidase in the whole cell lysates and medium of HeLa and HeLa/*POST1* KO cells. Values in HeLa cells were set to 1. Data are presented as mean ± SD (2 samples/trial; 3 independent trials). Unpaired two-tailed Student’s *t*-test, *P < 0.05, ***P < 0.001. (F) HeLa cells were transfected with the indicated siRNAs for 48 h, and stained with Lysotracker (red) for 30 min. Cells were then fixed and immunostained with the antibodies against LAMP1 (green). Scale bar, 10 μm. (G) HeLa cells were transfected with the indicated siRNAs for 48 h, fixed, stained with filipin (blue) and immunostained with the antibody against lyso-bis-phosphatidic acid (LBPA, green). Scale bar, 10 μm. (H) Quantification of filipin, Lysotracker and LBPA intensity in HeLa and HeLa/*POST1* KO cells shown in (F) and (G). Data are presented as mean ± SD (10 cells/trial; 3 independent trials). Unpaired two-tailed Student’s *t*-test. ***P < 0.001. (I) Schematic representation of POST1-promoted S1P maturation. POST1 accelerates the canonical autocleavage of S1P-B’/B at the C’/C sites, and the non-canonical autocleavage of S1P-A directly at the C’/C sites

SREBPs are among many membrane-bound transcription factors cleaved by S1P. We next sought to test where POST1 can affect proteolysis of other cellular substrates of S1P. Activating transcription factor 6 (ATF6) and cAMP response element–binding protein (CREB) 3 family members including CREB3L3 (also called CREBH) are ER-resident proteins that respond to stimuli by trafficking to the Golgi where they are sequentially cleaved by S1P and S2P (Ye et al., 2000; Zhang et al., 2006). ATF6 is a key player in unfolded protein response and CREBs regulate a wide array of genes involved in lipoprotein metabolism, collagen assembly, bone development and others. Figure 6A and 6B showed the processing of endogenous ATF6 and transfected CREB3L3 in WT and *POST1* KO cells exposed to thapsigargin for various periods. *POST1* KO cells had less amounts of the cleaved nuclear form of ATF6 (n-ATF6) and CREB3L3 (n-CREB3L3) than WT cells, suggestive of impaired cleavage of ATF6 and CREB3L3 in the absence of POST1.

S1P also regulates lysosomal biogenesis by cleaving the α/β-subunit precursor of N-acetylglucosamine (GlcNAc)-1-phosphotransferase, a key enzyme responsible for modifying newly synthesized lysosomal enzymes with mannose 6-phosphate (M6P) residues (Marschner et al., 2011). The α and β subunits are encoded by a single *GNPTAB* gene (Tiede et al., 2005). As shown in Fig. 6C, deficiency of *POST1* largely inhibited the cleavage of the α/β-subunit precursor to release α and β subunits. In line with this, the overall levels of M6P-modified proteins were reduced in cells lacking *POST1* or *S1P* (Fig. 6D). The intracellular abundance of α-mannosidase, an M6P-modified lysosomal enzyme, was significantly decreased but its secretion to the medium was greatly increased (Fig. 6E). We next examined the volume of lysosomes using the LAMP1 antibody and Lysotracker. Lysosome enlargement is a sign of dysfunction (te Vruchte et al., 2014; Xu et al., 2014). *POST1*-deficient cells had enlarged lysosomes as revealed by the LAMP1 and lysotracker staining (Fig. 6F). Defective lysosomal homeostasis in the absence of POST1 eventually caused massive accumulation of cholesterol and lyso-bis-phosphatidic acid (LBPA) in the cell (Fig. 6G and 6H), since lysosomal proteins such as NPC2 need M6P modification for lysosomal targeting (Wei et al., 2017).

## DISCUSSION

The impetus of the present study was to identify uncharacterized factors that regulate cholesterol metabolism. For this purpose, we performed a genome-scale, unbiased CRISPR/Cas9 KO screen coupled to the “cholesterol depletion-repletion-AmB selection” challenge (Fig. 1A), so that genes involved in LDL uptake and cholesterol trafficking to the PM were highly enriched. Our screen uncovered *C12ORF49*/*POST1*, the loss of which increased AmB resistance (Fig. 1C), attenuated SREBP target gene expression (Fig. 2A and 2B) and blocked SREBP processing (Fig. 3). Further examination showed that POST1 modulated SREBP signaling by accelerating generation (Fig. 4) and Golgi localization (Fig. 5) of mature S1P. In addition to SREBP activation, POST1-mediated S1P maturation is also critical for the cleavage of other S1P substrates including ATF6, CREB3L3 and the α/β-subunit precursor of GlcNAc-1-phosphotransferase (Fig. 6). These results set POST1 as a key determinant for S1P maturation and lipid metabolism. Based on these findings, we attribute the survival of *POST1*-deficient cells in the AmB screen to two reasons: 1) low LDLR expression as a result of impaired cleavage of SREBP2 (Figs. 2C and 3E); and 2) defective lysosomal cholesterol transport as a result of impaired cleavage of the α/β-subunit precursor of GlcNAc-1-phosphotransferase (Fig. 6C and 6G).

All nine members of the mammalian proprotein convertase family are synthesized as a zymogen and activated by autocatalytic cleavages of the N-terminal prodomain (Seidah and Prat, 2012). However, the self-processing of S1P is particularly complicated involving four identified cleavage sites (B’, B, C’ and C) and multiple cleavage steps, first at the B’/B sites and then the C’/C sites, yielding various forms of S1P with prodomain segments of different lengths bound non-covalently (Espenshade et al., 1999; Elagoz et al., 2002; da Palma et al., 2014). Contrasting to those in other proprotein convertases that function as an inhibitor, the prodomain of S1P is crucial for its folding, autoprocessing and proteolysis of substrates including SREBP2 (da Palma et al., 2014; da Palma et al., 2016). Another unique aspect of S1P is that it needs not to be completely processed to become enzymatically active, as S1P-B’/B can already cleave the SREBP2 precursor and activate the downstream signaling (Espenshade et al., 1999; da Palma et al., 2014). However, this is unlikely to be the case *in vivo* because S1P-B’/B resides in the ER and so does SREBP under cholesterol repletion conditions, in which the SREBP pathway is known to shut down. Instead, the B’/B forms may function both as the catalyst and substrate to give rise to the fully mature enzyme that can reach the Golgi and selectively deal with the translocated SREBP as a result of cholesterol depletion. Here, we demonstrate that POST1 is a S1P cofactor that promotes generation of S1P-C’/C, either from S1P-B’/B (canonical cleavage) or directly from S1P-A (non-canonical cleavage).

It is reported that all the S1P precursors reside in the ER and the mature S1P-C’/C resides in the Golgi (DeBose-Boyd et al., 1999). We demonstrate that POST1-EGFP-KDEL facilitates the production of S1P-C’/C, and that the S1P mutants that cannot be converted to the active form are mainly present in the ER (Fig. 5C and 5G). These data suggest that POST1 facilitates S1P-C’/C production in the ER and then they are transported to Golgi together. The mechanism by which POST1 contributes to S1P-C’/C production is unknown. However, this process is abolished by the D218A/H249A/S414A mutations (Fig. 4E), suggesting an absolute dependency on S1P enzymatic activity. Since autoprocessing at the C’/C sites is reported to occur in *trans* involving another S1P protein (Espenshade et al., 1999; da Palma et al., 2014), we speculate that POST1 may facilitate this intermolecular reaction by bringing two immature S1Ps in an optimal distance or orientation, so that one as the substrate can access the catalytic triad of another. It will also be interesting to examine whether POST1 affects autoprocessing of proprotein convertase subtilisin kexin 9 (PCSK9), which is classified as non-basic proprotein convertases along with S1P and, importantly, serves as an emerging drug target for hyperlipidemia and cardiovascular disease (Burke et al., 2017). The physiological regulator of POST1 is worth investigating as well.

During the preparation of our manuscript, C12ORF49 was identified as a key determinant of the SREBP pathway (Aregger et al., 2020 Bayraktar et al., 2020; Loregger et al., 2020). They all found that absence of *C12ORF49* reduced expression of SREBP target genes or impaired SREBP cleavage, which are consistent with our results. All their results can be explained by our finding that POST1 is required for S1P maturation. For example, Loregger et al. found that knockout of *C12ORF49* decreased SCAP protein level and caused SCAP relocation to the Golgi regardless of sterol levels. We believe that these phenotypes should be attributable to impairments in C12ORF49-mediated S1P maturation, and defective SREBP cleavage by S1P then prevents Golgi-to-ER transport of SCAP and causes SCAP degradation in lysosomes (Shao and Espenshade, 2014). Consistently, depletion of *POST1* or *S1P* similarly reduced SCAP level (Fig. S3A and S3B). As cholesterol depletion promotes ER-to-Golgi transport of SCAP and SCAP is degraded when SREBP cannot be efficiently cut by S1P, less SCAP was detected in *POST1*-KO cells under the sterol-depletion condition (Fig. S3A). If the reduced SCAP was the cause of impaired SREBP cleavage, the SREBP cleavage should be rescued by brefeldin A, an ER-Golgi protein trafficking inhibitor that disassembles and redistributes the Golgi complex into the ER (Sciaky et al., 1997). However, BFA largely restored the SREBP2 cleavage in the *SCAP* knockdown cells, with no effect on *POST1*- knockdown cells (Fig. S3C). So, less SCAP in POST1 KO cells is not the direct cause of impaired SREBP cleavage.

In summary, our study shows that POST1 is a cofactor of S1P. It promotes autocatalytic cleavage of S1P at the C’/C sites from immature S1P-A and S1P-B’/B. Through modulating S1P maturation, POST1 is critically involved in the processing of SREBP, ATF6, CREB3 family members and other S1P substrates.

## METHODS

### Reagents

Mevalonolactone (No. M4667), filipin (No. F9765), oleic acid (No. O1008), 25-HC (No. H1015) and anti-Flag M2 Affinity Gel (No. A2220) were from Sigma-Aldrich. LysoTracker Red DND-99 (No. L7528) and DAPI (No. D3571) were from Invitrogen. Blasticidin (No. 60218ES10) was from Yeasen. Endoglycosidase H (No. P0703S) and peptide N-glycosidase F (No. P0704S) were from New England Biolabs. Lipoprotein-deficient serum (LPDS, density >1.215 g/mL) was prepared from newborn calf serum by ultracentrifugation in our laboratory.

### Primary antibodies

The following antibodies were used in this study: mouse anti-ATF6 (Proteintech No. 66563-1-Ig), rabbit anti-α-subunit of GNPTAB (Abclonal Technology No. A15895), mouse anti-β-actin (Sigma No. A1978), mouse anti-clathrin heavy chain (BD Transduction Laboratories No. 610499), rabbit anti-CYP51A1 (Proteintech No. 13431-1-AP), rabbit anti-EGFP (Proteintech No. 50430-2-AP), mouse anti-FDFT1 (Santa Cruz Biotechnology No. sc-271602), mouse anti-Flag tag (Proteintech No. 66008-3-Ig), mouse anti-GAPDH (Proteintech No. 60004-1-Ig), mouse anti-GM130 (BD Transduction Laboratories No. 610823), mouse anti-lamin B1 (Proteintech No. 66095-1-Ig), mouse anti-lyso-bis-phosphatidic acid (LBPA) (Echelon Biosciences No. Z-PLBPA), rabbit anti-Myc tag (Proteintech No. 16286-1-AP), mouse anti-squalene epoxidase (Santa Cruz Biotechnology No. sc-271651). The polyclonal antibody against LDLR, the monoclonal antibody against HMGCR (A9), and the monoclonal antibody against SREBP2 (1D2) were prepared in our laboratory. The scFv (single-chain Fragment variable) M6P (mannose 6-phosphate) antibody was a kind gift from Dr. Thomas Braulke (University Medical Center Hamburg-Eppendorf, Germany). Primary antibodies were used at the dilution of 1:500 for immunofluorescent staining and 1:1,000 for immunoblotting.

### Plasmids

The coding sequences of *MBTPS1* (*S1P*) and *POST1* (*C12orf49*) were amplified from HeLa cells by standard PCR and inserted into pEGFP-N1, p3×Flag-CMV14 or pcDNA3.0 vectors to generate pCMV-POST1-EGFP, pCMV-S1P/POST1-3×Flag and pCMV-S1P/POST1, respectively. The sequence of POST1-3×Flag was sub-cloned from pCMV-POST1-3×Flag by standard PCR, and then inserted into the pLVX-IRES-Puro vector for lentivirus preparation. The sequence of 5×Myc tag was inserted into the C terminus of pCMV-S1P and pCMV-POST1 using site-directed mutagenesis via PCR. The sequences of KDAS and KDEL were inserted into the C terminus of pCMV-POST1-EGFP by overlap extension PCR. The mutant forms of S1P-EM (D218A/H249A/S414A), BM (R130E/R134E), CM (R163E/R164E/R183E/R184E) were prepared by site-directed mutagenesis. For pCMV-HA-1/HA-2-S1P-3×Flag, the sequence of HA tag (YPYDVPDYA) was inserted immediately after the sequence encoding the 30th and 152th amino acid of S1P protein in pCMV-S1P-3×Flag by site-directed mutagenesis. The coding sequence of *Creb3l3* (Addgene No. 99509) was sub-cloned into pcDNA3.0, and the sequence of 5×Myc tag was inserted into the N terminus *Creb3l3* to generate pCMV-5×Myc-Creb3l3. pCMV-GNPTAB (No. 78107), LentiCas9-Flag plasmid (No. 1000000049) and human CRISPR/Cas9 knockout pooled library (GeCKO v2) (No. 1000000048) were from Addgene.

### Cell culture

HeLa cells stably expressing POST-3×Flag (HeLa/POST-3×Flag) or stably expressing Cas9 (HeLa/Cas9-Flag) were generated by transducing HeLa cells with lentivirus expressing POST1-3×Flag or Cas9-Flag for 24 h. Cells were switched to Medium A (Dulbecco’s modified Eagle medium containing 10% fetal bovine serum, 100 units/mL penicillin and 100 μg/mL streptomycin sulfate) supplemented with 4 μg/mL puromycin or 10 μg/mL blasticidin for 4 days. Single cell colonies stably expressing POST-3×Flag were obtained by limiting dilution analysis. HeLa/*POST1* KO cells and HeLa/*S1P* KO cells were generated using the CRISPR-Cas9 technology (sgRNAs listed in Supplementary Material, Table S4) and isolated by limiting dilution analysis.

HeLa and HeLa/*POST1* KO cells were maintained in Medium A. HeLa/*S1P* KO cells were maintained in Medium A supplemented with 5 mg/mL cholesterol, 1 mmol/L mevalonate and 20 mmol/L oleic acid. The depletion medium was DMEM supplemented with 5% LPDS, 1 μmol/L lovastatin and 10 μmol/L mevalonate. 1 μg/mL 25-HC or 50 μg/mL LDL was added into the depletion medium if required. Cells were grown in a monolayer at 37 °C with 5% CO_2_.

### Genome-wide CRISPR/Cas9 screen coupled to AmB selection

HeLa/Cas9-Flag cells were transduced with lentivirus expressing a pooled GeCKO v2 library containing 65,383 sgRNAs targeting 19,050 human genes (3 sgRNAs per coding gene and 4 sgRNAs per microRNA) at 0.3 multiplicity of infection. Cells were incubated with Medium A containing 4 μg/mL puromycin for 4 days and then Medium A for another 3 days. A subpopulation of cells was collected to evaluate sgRNA target diversity. A greater than 300× library coverage was achieved. Transduced HeLa/Cas9-Flag cells were treated with the depletion medium for 16 h and then incubated with the depletion medium plus 50 μg/mL LDL and, if necessary, 2 μg/mL U18666A for 4 h. Cells were then incubated in the depletion medium supplemented with 50 μg/mL LDL and 300 µg/mL amphotericin B for 1 h. Cells were washed with PBS and incubated with Medium A for 4 days. A total of 5 rounds of “cholesterol depletion-repletion-AmB selection” was performed. The sgRNA inserts from surviving cells and those from transduced cells prior to the first round of selection were amplified and subjected to deep sequencing.

### Immunofluorescence

Cells grown on coverslips were fixed with 4% paraformaldehyde for 30 min and treated with 0.2% Triton X-100 in PBS for 5 min. Cells were washed with PBS and incubated with primary antibodies for 1 h at room temperature. After washing with PBS, cells were incubated with 3% bovine serum albumin (BSA) in PBS and appropriate secondary antibodies at a concentration of 1:1,000 for 1 h at room temperature. Cells were finally counterstained with 300 nmol/L DAPI in PBS for 5 min.

Confocal images were acquired by a Leica Biosystems SP8 laser scanning microscope. The contours of cell, Golgi and nuclei were outlined manually, and background-subtracted fluorescent intensity was quantified using ImageJ.

### Lysotracker and filipin staining

Cells were transfected with the indicated siRNA for 48 h and incubated with Medium A supplemented with 100 nmol/L Lysotracker for 30 min. Cells were then fixed and stained with 50 μg/mL filipin (prepared as 5 mg/mL stock solution in ethanol) in PBS for 1 h at room temperature.

### Immunoblotting analysis

Cells at a confluency of 80%–90% were harvested and homogenized with 120 μL of RIPA buffer supplemented with protease inhibitors. After centrifuging at 13,400 ×*g* for 10 min, supernatants were collected and protein concentration was determined using the BCA kit (ThermoFisher Scientific). If needed, 10 units/μL endoglycosidase H or 5 units/μL peptide N-glycosidase F was incubated with supernatants at 37 °C for 1 h. Supernatants were mixed with 4× loading buffer and boiled for 10 min. Proteins were resolved by SDS-PAGE and transferred to PVDF membrane. Blots were blocked with 5% BSA in TBS plus 0.075% Tween (TBST) and probed with primary antibodies overnight at 4 °C. After TBST wash, blots were incubated with secondary antibodies for 1 h at room temperature.

### Analysis of SREBP2 cleavage

Cells were transfected with the indicated siRNAs for 48 h and incubated with Medium A, the depletion medium or the depletion medium plus 25-HC for 16 h. For SREBP2 cleavage analysis using whole cell lysates, cells were treated with N-acetyl-leucinal-leucinal-norleucinal at a final concentration of 25 μg/mL for 1 h at 37 °C prior to harvesting.

For membrane fractionation, cells were incubated with buffer A (10 mmol/L HEPES/KOH, pH 7.6, 1.5 mmol/L MgCl_2_, 10 mmol/L KCl, 5 mmol/L EDTA, 5 mmol/L EGTA, 250 mmol/L sucrose) containing protease inhibitors on ice for 15 min. Cells were homogenized by passing through a 22G needle 30 times. After centrifuging at 1,000 ×*g* for 10 min, supernatants and pellets were separately collected. Supernatants were centrifuged at 20,000 ×*g* for 15 min at 4 °C, and pellet of this centrifugation (designed membrane fraction) was resuspended with SDS lysis buffer (10 mmol/L Tris-HCl, pH 6.8, 100 mmol/L NaCl, 1% (*w*/*v*) SDS, 1 mmol/L EDTA, 1 mmol/L EGTA) and mixed with 4× loading buffer. On the other hand, pellets were resuspended with buffer C (20 mmol/L HEPES/KOH, pH 7.6, 2.5% (*v*/*v*) glycerol, 1.5 mmol/L MgCl_2_, 0.42 mol/L NaCl, 1 mmol/L EDTA, 1 mmol/L EGTA) containing protease inhibitors and incubated at 4 °C for 1 h. Nuclear suspension was spun down at 13,200 rpm at 4 °C for 18 min, and supernatant of this centrifugation (designed nuclear extract) was collected and mixed with 4× loading buffer.

### Quantitative real-time PCR

Total RNA was extracted from HeLa cells transfected with the indicated siRNAs (listed in Supplementary Material, Table S5) or indicated knockout cells using TRIzol (Invitrogen No. 15596018). Equal amounts of RNA were used for cDNA synthesis followed by quantitative real-time PCR as previously described (37). The relative mRNA levels were calculated using the comparative CT method. Human *GAPDH* was used as the control. All qPCR primers are listed in Supplementary Material, Table S6. Gene expression in cells transfected with scramble siRNAs was defined as 1.

### Mass spectrometry

HeLa cells or HeLa/POST-3×Flag cells were homogenized in NP40 buffer (0.5% NP40 in PBS containing 5 mmol/L EDTA). After centrifuging at 2,000 ×*g* for 10 min, supernatants were collected and pre-cleared with protein A/G beads at 4 °C for 1 h. Mixtures were centrifuged at 1,000 ×*g* for 10 min, and supernatants were incubated with anti-Flag beads at 4 °C for 4 h. Beads were spun down and washed with NP40 buffer for 5 times, and proteins coupled to the beads were eluted by 0.1 mg/mL 3×Flag peptides. Eluents were collected and analyzed by liquid chromatograph-mass spectrometer. The intensity of protein present in HeLa/POST1-3×Flag cells divided by that in HeLa cells was defined as fold change. Proteins with fold change >1.5 were collected, and those detected in all three independent experiments were identified as POST1-interacting proteins.

### Statistical analysis

Data were expressed as means ± SD and analyzed by GraphPad Prism 6 software. Sample sizes, biological duplicates, statistical tests, *P* values were indicated in the figure legends. Statistical significance was set at *P* < 0.05.

## ELECTRONIC SUPPLEMENTARY MATERIAL

Below is the link to the electronic supplementary material.

Supplementary material 1 (PDF 628 kb)

Supplementary material 2 (MP4 1354 kb)

Supplementary material 3 (XLSX 639 kb)

Supplementary material 4 (XLSX 1665 kb)
