# Supplementary material for: POST1/C12ORF49 regulates the SREBP pathway by promoting site-1 protease maturation
Source: Protein Cell. 2020 Jul 14;12(4):279–96. doi: 10.1007/s13238-020-00753-3 (PMC8019017; doi:10.1007/s13238-020-00753-3)
Supplement: Supplementary file 1 — Supplementary material 1 (PDF 628 kb) [file 13238_2020_753_MOESM1_ESM.pdf]

Supplementary Information for

POST1/C12ORF49 regulates the SREBP pathway by promoting site-1 protease maturation

Jian Xiao[1], Yanni Xiong[1], Liu-Ting Yang[1], Ju-Qiong Wang[1], Zi-Mu Zhou[1], Le-Wei Dong[1], Xiong-Jie Shi[1], Xiaolu Zhao[1], Jie Luo[1,*], Bao-Liang Song[1,*]

[1]Hubei Key Laboratory of Cell Homeostasis, College of Life Sciences, Frontier Science Center for Immunology and Metabolism, Wuhan University, Wuhan 430072, China

[*]To whom correspondence may be addressed:
blsong@whu.edu.cn (B.-L. Song) and jieluo@whu.edu.cn (J. Luo)

This PDF file includes:

Figure S1 to S3

Tables S4 to S6

Other supplementary materials for this manuscript include the following:

Table S1. A full list of genes enriched in the cells survived 5 rounds of challenge.

Table S2. The transcriptome data of HeLa or HeLa/*POST1* KO cells exposed to the indicated medium.

Table S3. A full list of POST1-interacting proteins found in all three independent co-immunoprecipitation experiments coupled to tandem mass spectrometry.

Movie S1. A 3-D reconstructed movie showing the subcellular localization of POST1, SREBP2 and S1P.

Fig. S1

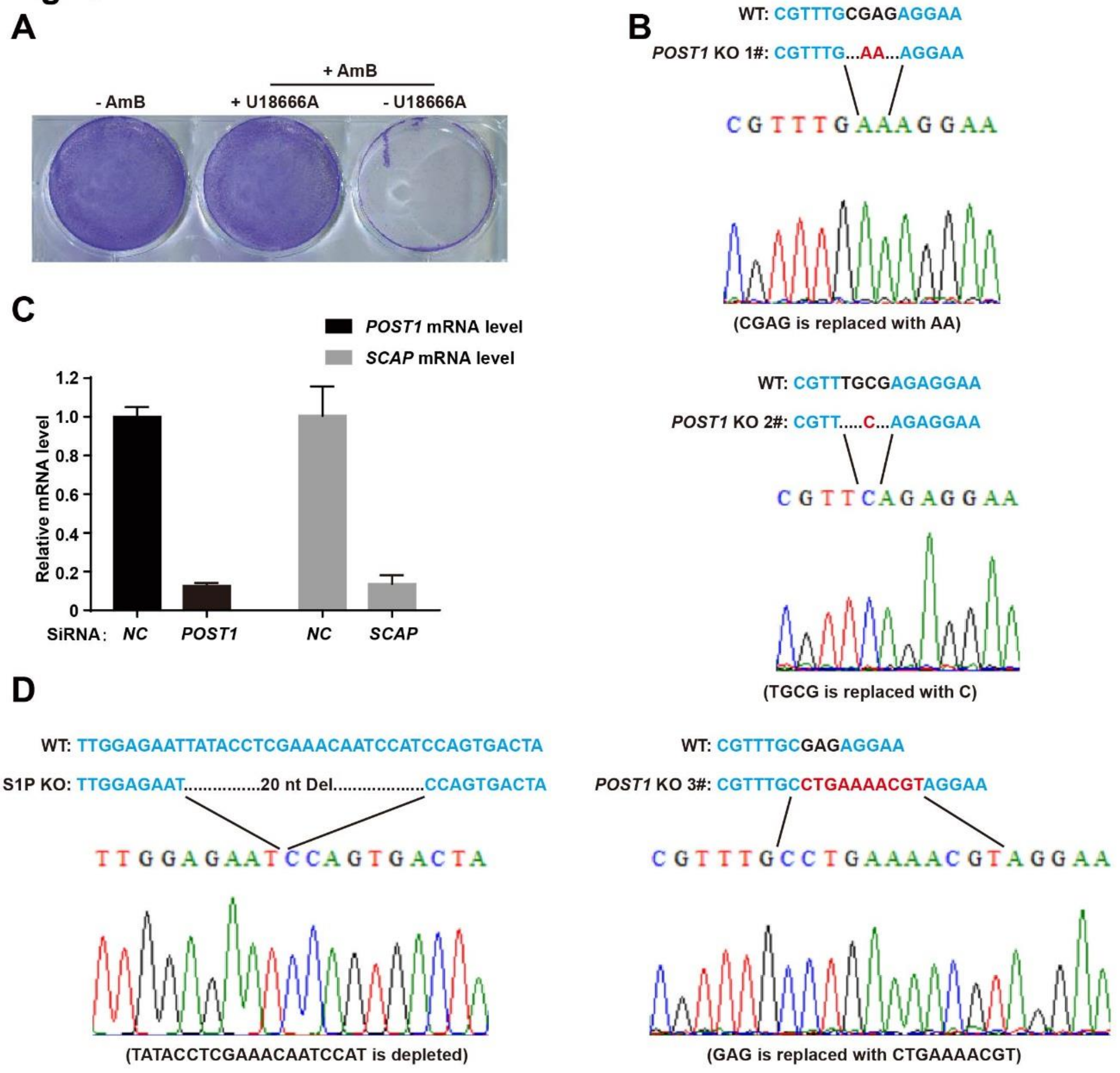

Figure S1. Validation of the AmB screen strategy, siRNA knockdown efficiency, and various knockout cells. (A) HeLa/Cas9-Flag cells were cultured in the cholesterol-depletion medium for 16 h and refed with LDL in the presence or absence of 2 μg/mL U18666A for 4 h. Cells were then treated with 300 μg/mL AmB for 1 h, fixed and stained with crystal violet. Cells with cholesterol depletion and repletion challenge but no exposure to U18666A or AmB were used as a control. (B) Sanger sequencing analysis of three independent clones of HeLa/*POST1* KO cells bearing different mutations in the exon 3 of the *POST1* gene. (C) The knockdown efficiency of siRNAs targeting *POST1* and *SCAP*. HeLa cells were transfected with scrambled siRNA, *POST1* or *SCAP* siRNA for 48 h and harvested for quantitative real-time PCR analysis. The relative mRNA level of *POST1* or *SCAP* was normalized to that of *GAPDH* and presented as mean ± SD (n = 3). (D) Sanger sequencing analysis of HeLa/*S1P* KO cells bearing a frameshift mutation in the exon 3 of the *S1P* gene.

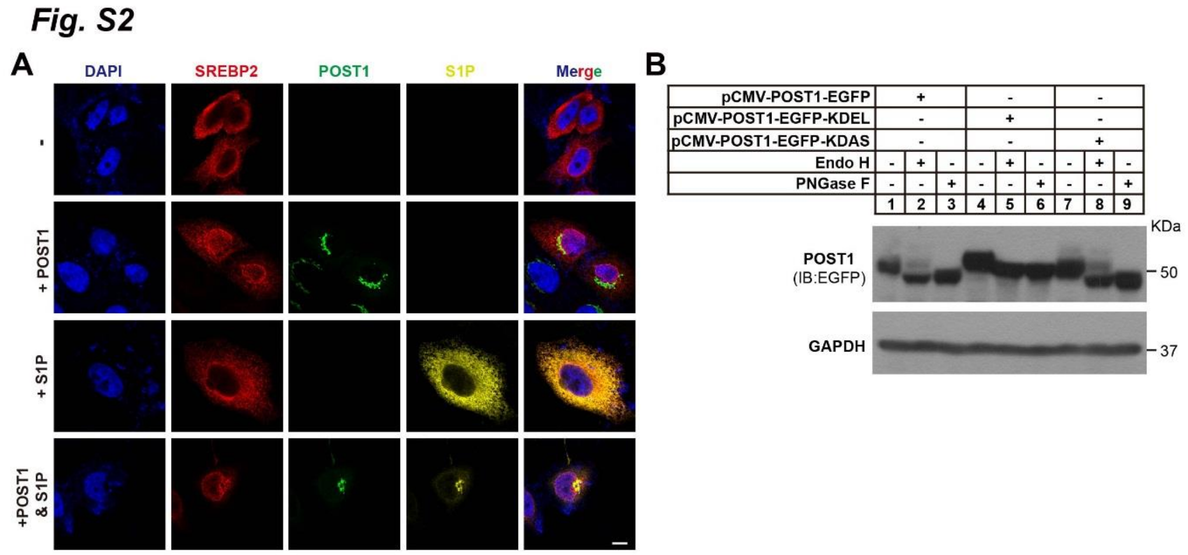

Figure S2. Localization of POST1, S1P and SREBP2 and glycosidase digestion assay of POST1 variants. (A) HeLa cells were transfected with pCMV-SREBP2-Flag (red), pCMV-POST1-EGFP (green), and pCMV-S1P-Myc (Yellow) as indicated for 48 h. Cells were fixed and immunostained with the antibodies against Flag and Myc. The nuclei were counterstained with DAPI (blue). Scale bar, 10 µm. (B) HeLa cells were transfected with pCMV-POST1-EGFP, pCMV-POST1-EGFP-KDEL and pCMV-POST1-EGFP-KDAS as indicated for 48 h and harvested. Lysates were treated with 10 units/µL Endo H or 5 units/µL PNGase F as indicated prior to immunoblotting.

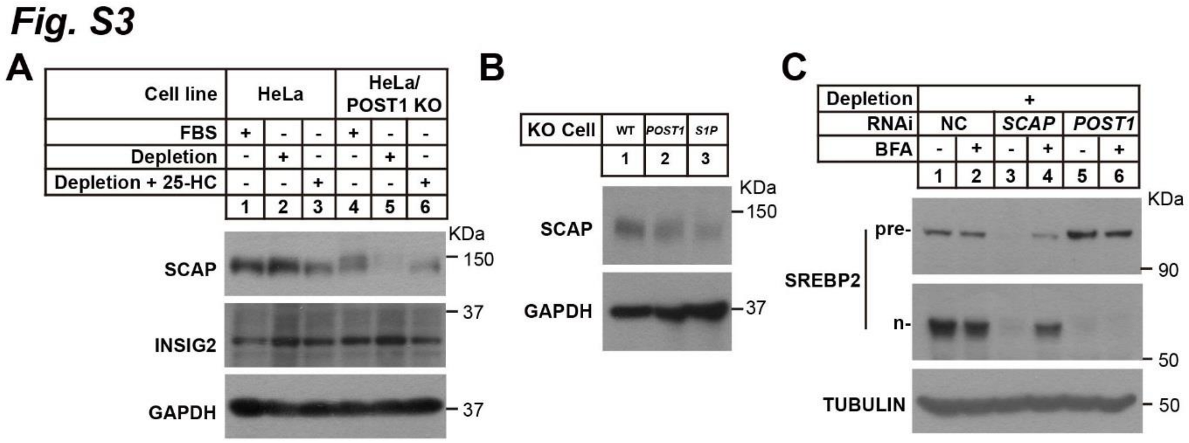

Figure S3. BFA fails to rescue impaired SREBP2 cleavage in *POST1*-knockdown cells. (A) Immunoblot analysis of HeLa, HeLa/*POST1* KO and HeLa/*S1P* KO cells under different culture conditions using antibodies against SCAP, INSIG2 and GAPDH. (B) Immunoblot analysis of SCAP protein level in HeLa and HeLa/*POST1* KO cells grown under FBS conditions. (C) HeLa cells transfected with the indicated siRNA for 48 h, incubated with the depletion medium for 16 h, and treated with 1 μg/mL brefeldin A (BFA) for 5 h. Cells were harvested and immunoblotted with antibodies against SREBP2 and TUBULIN.

Table S4. Primers used for generation of HeLa/*POST1* KO and HeLa/*S1P* KO cells.

	Forward (5' - 3')	Reverse (5' - 3')
POST1 KO primer	CACCGTTTCAGGCTACGTTTGCGAG	AAACCTCGCAAACGTAGCCTGAAAC
S1P KO primer	CACCGTCACTGGATGGATTGTTTCG	AAACCGAAACAATCCATCCAGTGAC

Table S5. SiRNAs used in this study.

	Sequence (5' - 3')
siRNA for *POST1*	CCTACCTTGTGGTGGTTAT
siRNA for *SCAP*	CCAGGTTCATGACCATAAT

Table S6. Primers used for quantitative real-time PCR analysis in this study.

Gene name	Forward (5' - 3')	Reverse (5' - 3')
POST1	ACTTGGGCAATAGCAGTCGTC	GCAAACGTAGCCGAGTTCAT
SCAP	TATCTCGGGCCTTCTACAACC	GGGGCGAGTAATCCTTCACA
HMGCS1	GACTTGTGCATTCAAACATAGCAA	GCTGTAGCAGGGAGTCTTGGTACT
HMGCR	CAAGGAGCATGCAAAGATAATCC	GCCATTACGGTCCCACACA
FDFT1	CCACCCCGAAGAGTTCTACAA	TGCGACTGGTCTGATTGAGATA
SQLE	TGTCGCCACCGAAACGG	ATATTGGTTCCTTTTCTGCGCCTC
LSS	GCACTGGACGGGTGATTATGG	TCTCTTCTCTGTATCCGGCTG
CYP51A1	CCTGGCTCTTACCAGGTTGG	GTCTGCGTTTCTGGATTGCC
ACC1	GAGCAAGGGATAAGTTTGAG	AGGTGCATCTTGTGATTAGC
SCD1	TCTAGCTCCTATACCACCACCA	TCGTCTCCAACTTATCTCCTCC
FASN	ACACAGTCACCATCTCGG	CAAACACACCCTCCTTCCT
INSIG1	CCTGGCATCATCGCCTGTT	AGAGTGACATTCCTCTGGATCTG
INSIG2	CTTGATGATTCGAGGAGTAGTGC	CAGGTGGAAAGAGCGTCACAT
GAPDH	GGAGCGAGATCCCTCCAAAAT	GGCTGTTGTCATACTTCTCATGG

Movie S1. A 3-D reconstructed movie showing the subcellular localization of POST1, SREBP2 and S1P. HeLa cells were co-transfected with pCMV-SREBP2-Flag (red), pCMV-POST1-EGFP (green) and pCMV-S1P-Myc (yellow) for 48 h. Cells were fixed and immunostained with the antibody against Flag and Myc. The nucleus was counterstained with DAPI (blue). A 3-D image stacks in the Z-axis were acquired by a Leica SP8 confocal microscopy and processed with 3-D rendering by the Leica X software to generate the movie.
